# Spontaneous uterine rupture in the second trimester with fetal Cyclopia at a resource-limited setting: A case report

**DOI:** 10.1016/j.ijscr.2025.111228

**Published:** 2025-03-27

**Authors:** Mohamed Ahmed Abdillahi, Ahmed Abdi Aw Egge, Chaltu Resassa Shashe, Kenzu Bedru Hussen, Abdisalam Aden Dahir, Abdisalam Hassan Muse

**Affiliations:** a Al-Hayat Teaching and Research Hospital, Amoud University, Amoud Valley, Borama 25263, Somaliland, Somalia; b School of Medicine and Surgery, College of Health Science, Amoud University, Amoud Valley, Borama 25263, Somaliland, Somalia; c Department of Anaesthesia, School of Nursing and Midwifery, College of Health Science, Amoud University, Amoud Valley, Borama 25263, Somaliland, Somalia; d Research and Innovation Centre, Amoud University, Amoud Valley, Borama 25263, Somaliland, Somalia

**Keywords:** Uterine rupture, Spontaneous uterine rupture, Second trimester, Cyclopia, Case report, Somaliland

## Abstract

**Introduction and importance:**

Spontaneous uterine rupture in the second trimester is a rare and life-threatening obstetric emergency, typically associated with pre-existing risk factors. This case presents a unique instance of spontaneous uterine rupture in a multiparous woman without typical risk factors, highlighting the challenges of diagnosis and management in a resource-limited setting. The associated fetal cyclopia adds to the rarity of the case and contributes to the existing literature on uterine rupture. This report demonstrates the need for high clinical suspicion and prompt intervention, even in the absence of usual risk factors.

**Case presentation:**

A 30-year-old, gravida 7, para 6 female presented with a two-day history of abdominal pain, vomiting, and subjective fever. The patient progressed to hemodynamic instability, vaginal bleeding, and loss of consciousness within a few hours of admission.

**Clinical discussion:**

The main diagnosis was spontaneous uterine rupture with hemoperitoneum and a non-viable fetus with cyclopia. The therapeutic interventions included emergency exploratory laparotomy, evacuation of the hemoperitoneum, and subtotal hysterectomy. The patient recovered well and was discharged on postoperative day five.

**Conclusion:**

This case underscores the unpredictable nature of uterine rupture and the importance of maintaining a high index of suspicion for this diagnosis, even in the absence of typical risk factors, particularly in resource-limited settings. Prompt surgical intervention and appropriate postoperative care are essential for favorable maternal outcomes.

## Highlights

• Rare case of spontaneous uterine rupture in the second trimester *without* typical risk factors.
• Fetal cyclopia adds to the rarity of this obstetric emergency.
• Highlights diagnostic and management challenges in a resource-limited setting
• Emphasizes the need for high clinical suspicion even in the absence of usual risk factors.
• Successful maternal outcome achieved through prompt surgical intervention.

## 1 Introduction

Uterine rupture, a full-thickness disruption of the uterine wall, is a grave obstetric complication that carries significant risks of maternal and fetal morbidity and mortality [1,2]. The incidence of uterine rupture varies geographically, with higher rates reported in low-income countries (1 in 100 to 500 deliveries) compared to high-income settings (1 in 3000 to 5000 deliveries) [1].

Spontaneous uterine rupture (SUR) in the second trimester, without prior uterine surgery or known anomalies, is exceedingly rare [3,4]. This is often linked to conditions such as trophoblastic disease or abnormal placentation (placenta accreta, increta, percreta) [4]. Traditional risk factors include maternal age, high parity, fetal macrosomia, shoulder dystocia, and prior induced abortions [5]. This case is unique as the patient had no known history of these typical risk factors and the rare association with fetal Cyclopia.

Management often necessitates surgical intervention, ranging from uterine repair to hysterectomy. In cases where the fetus is pre-viable, management decisions are complex. This case report, originating from a teaching and research hospital affiliated with Amoud University in Borama, Somaliland, highlights the diagnostic and therapeutic challenges of managing spontaneous uterine rupture in a resource-limited setting.

## 2 Patient information

The patient was a 30-year-old, gravida 7, para 6, multiparous Somalian woman. Her ethnicity was Somali and her occupation was a homemaker. There is no data available on her BMI and the hand dominance was not recorded.

The patient presented to the emergency department of Al-Hayatt Teaching and Research Hospital in the late evening with a two-day history of sudden onset lower abdominal pain, associated with nausea, vomiting, and subjective fever. She was not brought in by ambulance and walked to the emergency room from her nearby house.

The patient had a history of six previous pregnancies, resulting in three live births and three stillbirths, all via spontaneous vaginal delivery. The deliveries all took place in a rural setting. The causes of the previous three stillbirths were not documented and remain unknown. The patient did not report any specific complications during those pregnancies, but she delivered in a rural setting with limited access to prenatal care. She had no history of abortions, cesarean sections, assisted deliveries, dilation and curettage (D&C), or intrauterine device (IUD) insertions.

Given the resource-limited setting, advanced diagnostic tools such as CT scans or MRI were unavailable. The initial differential diagnosis included placental abruption, ectopic pregnancy, acute appendicitis, and pyelonephritis.

She reported no prior medical disorders, surgeries, current or past medications, or relevant family history. She did not report any bowel changes, headache, jaundice, vaginal bleeding, and urinary symptoms or significant psychosocial issues. The patient was not a smoker. Data about her accommodation type, walking aids etc. was not collected. The work has been reported in line with the SCARE criteria [6].

## 3 Clinical findings

On initial examination, her vital signs were relatively stable, with a pulse of 90 beats/min and a blood pressure of 110/80 mmHg. She appeared mildly distressed. Abdominal examination revealed a symphysis-fundal height of 25 cm, a single fetus in a longitudinal lie with a cephalic presentation, and a fetal heart rate of 140 beats/min via Doppler. She exhibited tenderness in the epigastric and lower abdominal areas, but no signs of peritonitis. Pelvic examination revealed a closed cervical os with no bleeding or vaginal discharge. No clinical photographs are available. Unfortunately, the bedside ultrasound images are unavailable for inclusion in this report.

## 4 Timeline

The patient's clinical course began on Day 1 in the afternoon when she presented with a two-day history of abdominal pain, vomiting, and subjective fever, leading to her admission to the Obstetric ward for observation. By the early hours of Day 2, approximately six hours after admission, her condition deteriorated rapidly. She experienced loss of consciousness, tachycardia, hypotension, pallor, abdominal distension, and vaginal bleeding. Resuscitation was immediately initiated, including intravenous fluid administration and one unit of blood transfusion. On the same day, an emergency laparotomy was performed, which revealed a uterine rupture and a stillborn fetus with cyclopia. Later that same afternoon, the patient was transferred to the recovery room with stable vital signs and then to the postnatal ward for ongoing care. Her recovery progressed, and she was discharged home on Day 7.

## 5 Diagnostic assessment

The diagnostic process involved several methods. Physical examination revealed abdominal tenderness which later progressed to signs of peritonitis, accompanied by pallor and hypotension. Laboratory tests at admission showed a hemoglobin level of 14.5 g/dl, a white blood cell count of 4.5 (10^9/L), a platelet count of 203 (10^9/L), and a creatinine level of 0.6 mg/dl. The patient's blood group was O Rh positive, with normal electrolytes, urine analysis, and blood glucose levels. A subsequent drop in hemoglobin to 13.5 g/dl was noted after admission and a further decrease to 11.5 g/dl after surgery. Bedside ultrasound imaging indicated a single cephalic non-viable intrauterine pregnancy with a single dilated ventricle and polyhydramnios.

**Fig. 1:**
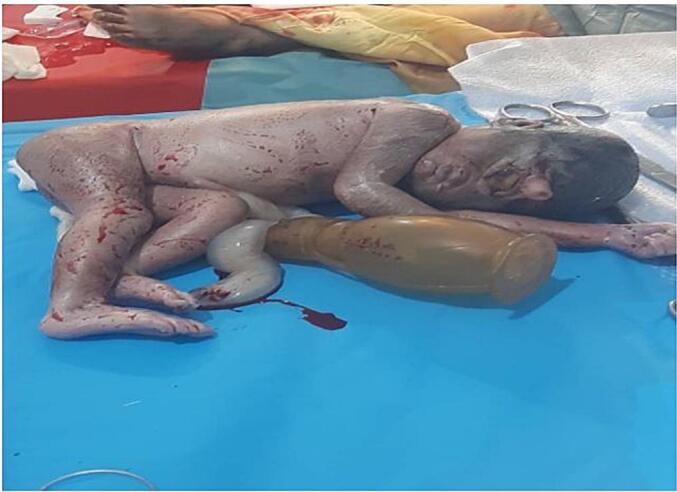
A photograph of the stillborn fetus showing features of cyclopia - A single eye in a single orbit with a tube-like structure above the eye.

It also revealed a large collection of free fluid in the peritoneum and a defect on the posterior uterine wall, with an anteriorly located placenta. There were some diagnostic challenges, primarily limited access to advanced imaging techniques such as CT scans or MRI. The initial non-specific symptoms also contributed to a delay in suspecting uterine rupture. Cultural factors, however, did not contribute to any diagnostic challenges. Differential diagnoses considered included placental abruption, ectopic pregnancy, acute appendicitis, and pyelonephritis Fig. 1. The rapid deterioration of the patient's condition combined with the findings on ultrasound led to a strong suspicion of uterine rupture. Prognostic characteristics were not directly applicable in this case, although it's noted that fetal cyclopia is associated with a poor prognosis. No radiological images were taken, and no pre-operative histopathological assessments were conducted.

## 6 Therapeutic intervention

Prior to surgical intervention, the patient was actively resuscitated with intravenous fluids and blood transfusions to address hypovolemia and hypotension. She did not require admission to the Intensive Care Unit (ICU). The primary therapeutic intervention was an emergency surgical procedure aimed at controlling the hemorrhage and preventing further complications resulting from the uterine rupture. Due to the extensive nature of the rupture and its extension down to the vagina, a subtotal hysterectomy was deemed necessary, along with the removal of the left fallopian tube and ovary. The left fallopian tube and ovary were removed due to significant adhesions and inflammation observed during the surgery, raising concerns about potential long-term complications if left in situ. The patient also received pharmacological treatments including analgesics, antibiotics, and further blood transfusions as required to manage the pain, prevent infection, and replace lost blood volume.

**Fig. 2:**
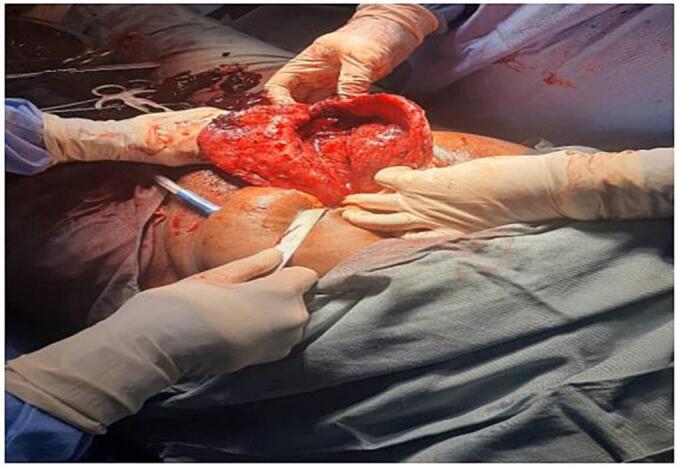
Intraoperative photograph showing the extent of uterine rupture from fundus to the vagina.

During the peri-operative period, the patient was placed under general anaesthesia, which was administered by two nurse anaesthetists. A midline infra-umbilical exploratory laparotomy was performed with the patient in a supine position. Surgical preparation of the skin was done using iodine solution, and standard surgical instruments and sutures were used for the procedure. The surgery was performed by a team consisting of a senior gynaecologist, a senior general surgeon, a family physician, and a general practitioner. This surgical approach was not new, and all members of the team were trained and experienced in performing standard hysterectomies. Initially, there was an attempt to repair the uterine defect, but due to the extent of the damage, this was not feasible. Intra-operative photographs documenting the extent of the rupture are included in Fig. 2.

Due to the extensive nature of the rupture and its extension down to the vagina, a subtotal hysterectomy was deemed necessary. Uterine repair was considered but deemed not feasible due to the extent of the damage and the risk of further complications. The left fallopian tube and ovary were removed due to significant adhesions and inflammation observed during the surgery, raising concerns about potential long-term complications if left in situ.

A histopathological assessment of the uterus was not performed. This surgery is not considered novel or a first-in-human procedure. Post-operatively, the patient received standard care, including pain management, antibiotic therapy, and careful monitoring for any signs of complications. She was subsequently admitted to the postnatal ward for continued care and observation. The patient received counselling regarding the implications of the hysterectomy on future fertility. Due to resource limitations, comprehensive psychological support was not readily available, but the patient was advised to seek further care if needed and was given information on local support groups.

## 7 Follow-up and outcomes

Clinician-assessed outcomes included stable vital signs, normal temperature, improved general condition, and return to normal activity. Patient-reported outcomes at the follow-up were good mood and overall well-being. Post-operative follows up time was 4 weeks. There are no photographs or radiological images available for the follow-up period.

The patient was scheduled for a follow-up appointment 4 weeks post-surgery to assess her recovery and mental health. She did not require ongoing diagnostic testing. No surveillance is required. The patient adhered well to post-operative care instructions and tolerated them well.

No complications were observed. No information was provided on blood loss. Operative time was not mentioned in the original case. The wound healing was uneventful, with no signs of infection or dehiscence. There were no re-exploration/revision surgeries, and no mortality was reported within 30 days of the operation.

## 8 Discussion

This case report's primary strength lies in its detailed documentation of a rare obstetric emergency: spontaneous uterine rupture (SUR) in the second trimester, occurring in the absence of typical risk factors and complicated by the presence of fetal cyclopia, all within a resource-limited setting [1]. However, several limitations must be acknowledged. These include the lack of access to advanced diagnostic tools such as CT scans or MRI, which could have provided more comprehensive pre-operative information [2]. Additionally, there was a lack of detailed pre- and post-operative data and limited documentation of the patient's specific contraindications for a subtotal hysterectomy.

The absence of a histopathological assessment of the uterus and further genetic investigations for the fetus also represents a limitation to the complete understanding of this case [3]. The existing literature describes uterine rupture as commonly associated with a history of prior cesarean sections, uterine abnormalities, and other obstetric procedures [4,5]. In contrast, this case presented multiparity as the only identifiable risk factor.

The case also highlights the rarity of SUR occurring spontaneously in the second trimester, without prior uterine surgeries or other typical risk factors. While SUR typically occurs at the fundus, and lower uterine segment ruptures during labor, this case is unique due to the abnormal extension of the rupture from the fundus to the vagina [6]. This case report, therefore, adds to the limited body of literature on similar occurrences and further explores the potential link between fetal cyclopia and uterine rupture, though the association might be coincidental [[7], [8], [9]]. The conclusions drawn from this case are based on the patient's clinical presentation, the diagnostic findings, and the observed surgical findings.

The patient's rapid deterioration underscored the need for prompt clinical recognition and intervention [10,11]. The primary takeaway lesson is that clinicians must remain aware of the possibility of spontaneous uterine rupture in the second trimester, even in the absence of typical risk factors. They should maintain a high index of suspicion for uterine rupture even in cases of pregnant women presenting with seemingly common gastrointestinal symptoms, particularly in resource-constrained settings, where resources for rapid diagnosis and management may be limited [12,13].

While this case presents a unique association between uterine rupture and fetal cyclopia, the possibility of a coincidental relationship cannot be ruled out. The existing literature offers limited insight into a direct pathophysiological link between these two conditions. Further research is needed to explore potential genetic or environmental factors that may contribute to both uterine rupture and fetal cyclopia. Other reported cases of second-trimester uterine rupture in unscarred uteri often involve similar management strategies, with surgical intervention being the primary approach. However, the specific choice between uterine repair and hysterectomy depends on the extent of the rupture and the patient's overall condition.

## 9 Limitations

This case report is limited by the single case study design, which restricts the generalizability of the findings. The unavailability of histopathological analysis of the uterus and advanced imaging techniques such as CT scans or MRI further limits our understanding of the underlying pathology. The limited follow-up data also restricts our ability to assess long-term outcomes and potential complications. Despite these limitations, this case provides valuable insights into the management of spontaneous uterine rupture in a resource-limited setting.

## 10 Conclusion

This case highlights the importance of considering spontaneous uterine rupture in the differential diagnosis of abdominal pain in pregnant women, even in the absence of typical risk factors. Prompt surgical intervention is crucial for maternal survival, particularly in resource-limited settings. While this case highlights the importance of considering spontaneous uterine rupture in the differential diagnosis, further research is needed to establish the specific risk factors and optimal management strategies for this rare condition, particularly in resource-limited settings. The lack of histopathological confirmation and limited follow-up data warrant a cautious interpretation of our findings. More research is needed to establish the risk factors and management strategies for second-trimester uterine rupture in unscarred uteri.

Figures.

## Author contribution

All authors contributed equally to this manuscript.

## Consent

Written informed consent was obtained from the patient for publication of case details and accompanying images.

## Ethical approval

This case report was approved by the Al-Hayat Teaching and Research Referral Hospital (Ref: AU-AH-B-ERC-2024). Furthermore, this case report has been prepared in accordance with the SCARE (Surgical CAse REport) guidelines [6,14].

## Guarantor

Abdisalam Hassan Muse.

## Patient perspective

Not applicable. The patient perspective was not specifically sought.

## Funding

The authors did not receive any funding for this work.

## Conflict of interest statement

The authors declare no conflicts of interest.
